# Vision, Perception, and Attention through the Lens of Microsaccades: Mechanisms and Implications

**DOI:** 10.3389/fnsys.2015.00167

**Published:** 2015-12-02

**Authors:** Ziad M. Hafed, Chih-Yang Chen, Xiaoguang Tian

**Affiliations:** ^1^Physiology of Active Vision Laboratory, Werner Reichardt Centre for Integrative Neuroscience, University of TuebingenTuebingen, Germany; ^2^Graduate School of Neural and Behavioural Sciences, International Max-Planck Research School, University of TuebingenTuebingen, Germany

**Keywords:** fixational eye movements, microsaccades, covert visual attention, perceptual stability, superior colliculus, frontal eye fields

## Abstract

Microsaccades are small saccades. Neurophysiologically, microsaccades are generated using similar brainstem mechanisms as larger saccades. This suggests that peri-saccadic changes in vision that accompany large saccades might also be expected to accompany microsaccades. In this review, we highlight recent evidence demonstrating this. Microsaccades are not only associated with suppressed visual sensitivity and perception, as in the phenomenon of saccadic suppression, but they are also associated with distorted spatial representations, as in the phenomenon of saccadic compression, and pre-movement response gain enhancement, as in the phenomenon of pre-saccadic attention. Surprisingly, the impacts of peri-microsaccadic changes in vision are far reaching, both in time relative to movement onset as well as spatial extent relative to movement size. Periods of ~100 ms before and ~100 ms after microsaccades exhibit significant changes in neuronal activity and behavior, and this happens at eccentricities much larger than the eccentricities targeted by the microsaccades themselves. Because microsaccades occur during experiments enforcing fixation, these effects create a need to consider the impacts of microsaccades when interpreting a variety of experiments on vision, perception, and cognition using awake, behaving subjects. The clearest example of this idea to date has been on the links between microsaccades and covert visual attention. Recent results have demonstrated that peri-microsaccadic changes in vision play a significant role in both neuronal and behavioral signatures of covert visual attention, so much so that in at least some attentional cueing paradigms, there is very tight synchrony between microsaccades and the emergence of attentional effects. Just like large saccades, microsaccades are genuine motor outputs, and their impacts can be substantial even during perceptual and cognitive experiments not concerned with overt motor generation *per se*.

## Introduction

Ever since the advent of modern-day systems neuroscience, the use of awake, behaving subjects (such as human and non-human primates), combined with quantitative analysis of neuronal and behavioral data, has provided us with unprecedented access to internal brain processes “as they happen” (i.e., during behavior). However, in the case of vision, eye movements posed a serious technical challenge, since eye movements translate images of stationary stimuli across the retina (and therefore across many retinotopically organized visual areas). As a result, “gaze fixation” was, and still is, usually enforced in experiments, with the logic being that when subjects fix their gaze, visual stimulation of the retina due to eye movements would now be experimentally “eliminated”, allowing scientists to use presumably “identical” stimuli and to investigate modulations in representing these stimuli that may be related to visual analysis, perceptual interpretation, decision making, or cognitive processing. However, even during gaze fixation, tiny eye movements continuously occur (Barlow, 1952), and it is now increasingly evident that these eye movements are not random, and therefore not amenable to being “averaged out” in analyses (Rolfs, 2009; Hafed, 2011; Poletti and Rucci, 2015). Moreover, at least some of these eye movements have dramatic extra-retinal impacts on visual representations, even in the far periphery (Leopold and Logothetis, 1998; Hafed and Krauzlis, 2010; Chen et al., 2015). Coupled with theoretical and experimental studies on the potential implications of these eye movements on input sensory stream statistics (Gur et al., 1997; Rucci et al., 2007; Kuang et al., 2012; Snodderly, 2014; Poletti and Rucci, 2015; Rucci and Victor, 2015), this suggests that the mere act of moving the eyes by the equivalent of only a handful of retinal photoreceptors can have real and measurable impacts on neuronal activity and behavior. In this review, we highlight recent advances in our understanding of the extent of these impacts for the specific case of microsaccades, the most well-studied and well-discussed sub-type of fixational eye movements to date (Collewijn and Kowler, 2008; Rolfs, 2009; Hafed, 2011; Snodderly, 2014; Poletti and Rucci, 2015).

Microsaccades are small saccades that occur intermittently during fixation. These eye movements have experienced a dramatic resurgence in research over the past ~15 years, and this resurgence has been due to a variety of factors (Hafed, 2011), including the advent of awake, behaving monkey neurophysiology in the preceding 20–30 years, the development of accessible non-invasive eye tracking methods, and the development of novel ideas on how microsaccades might interact with perception and cognition. As a result of such resurgence, microsaccades are now viewed differently from how they were predominantly regarded in the previous century. By the end of the last century, microsaccades were generally considered to be a curiosity that is inconsequential for the interpretation of experiments due to their tiny size. Specifically, with large peripheral receptive field (RF) sizes in many areas of the visual system, small shifts in the retinal image due to microsaccades were considered to be minimal and random. However, in the current century, several lines of evidence have emerged on the neuronal mechanisms associated with microsaccades. Besides filling important gaps in our understanding of these mechanisms, such evidence has also pointed towards the existence of microsaccadic influences that go beyond simple retinal-image shifts. Such evidence is increasingly challenging the view that microsaccades are inconsequential for the interpretation of a variety of experiments involving gaze fixation.

**Figure 1:**
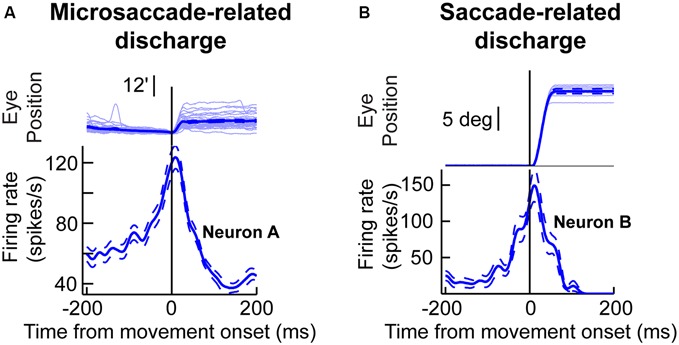
**Microsaccade and saccade-related discharge in the primate superior colliculus (SC). (A)** Neuronal activity from a sample SC neuron around the time of microsaccade generation. The upper traces show eye position traces aligned (in terms of the *x*-axis) on the onset of microsaccades smaller than ~0.2 degrees in amplitude (note that the vertical *y*-axis position of all eye traces was aligned at microsaccade onset to facilitate inspection of the movement amplitudes). The bottom panel shows the firing rate of the neuron before, during, and after the microsaccades shown in the upper panel. As can be seen, the neuron exhibited buildup of activity leading to movement onset, a phasic burst during the movements, and then a return to baseline afterwards. **(B)** Activity of a saccade-related neuron from another portion of the SC map. This neuron was tuned for saccades larger than ~10 degrees in amplitude. Whenever the monkey made a large saccade of this amplitude, the neuron showed classic saccade-related discharge that is similar to the microsaccade-related discharge in **(A)**. This figure was adapted with permission from Hafed (2011).

One key to recasting the old prevailing view of microsaccades, which is particularly relevant for this review, came from insights into their generation mechanisms. It was found that the midbrain superior colliculus (SC) plays a causal role in microsaccade generation (Hafed et al., 2009; Goffart et al., 2012; Hafed and Krauzlis, 2012). Importantly, microsaccade generation mechanisms in the SC are similar to those for large saccades. Neurons with movement fields “tuned” for a given set of microsaccade amplitudes and directions exhibit pre-movement buildup of activity, a phasic burst during the microsaccade, and a gradual return to baseline (Figure 1A). Such movement-related discharge is virtually indistinguishable from saccade-related discharge of SC neurons with movement fields tuned for large saccades (Figure 1B). From the perspective of this review, this is particularly interesting: peri-saccadic changes in vision that accompany large saccades have time courses that are similar to the time courses of saccade-related discharge in the SC (Figure 1B), and these peri-saccadic changes are at least partially mediated by SC activity (Sommer and Wurtz, 2002, 2006; Phongphanphanee et al., 2011); thus, the existence of microsaccade-related discharge in the form shown in Figure 1A hints that there can also be similar peri-microsaccadic changes in vision. In what follows, we describe recent discoveries pointing in that direction. From a broader perspective, these discoveries are relevant for a variety of experiments on vision, perception, and attention because microsaccades inescapably occur in these experiments (Hafed and Clark, 2002; Engbert and Kliegl, 2003; Rolfs et al., 2008; Rolfs, 2009; Hafed, 2011; Hafed and Ignashchenkova, 2013; Snodderly, 2014; Poletti and Rucci, 2015). We finish this review by demonstrating examples of this idea for the case of the role of microsaccades in covert visual attention.

## Microsaccadic Suppression

Early neurophysiological studies on the impacts of microsaccades on vision have concentrated on early sensory areas in the visual system (Gur and Snodderly, 1987, 1997; Gur et al., 1997; Bair and O’Keefe, 1998; Leopold and Logothetis, 1998; Martinez-Conde et al., 2000, 2002, 2013; Snodderly et al., 2001; Kagan et al., 2008; Bosman et al., 2009; Herrington et al., 2009). A primary observation from all of these studies has been that the lateral geniculate nucleus (LGN), primary visual cortex (V1), as well as visual areas V2 and V4 all exhibit, to varying degrees of strength, post-microsaccadic *enhancement* of firing rates. Moreover, area MT is sensitive to the retinal-image motion caused by microsaccades (Bair and O’Keefe, 1998). Even though the origins of post-microsaccadic enhancement of neuronal activity are not fully known, a very likely component of these origins is the retinal-image “refreshing” that is caused by global translations of images over retinal photoreceptors (Roska and Werblin, 2003). An extra-retinal component for such post-microsaccadic enhancement of firing rates has also been suggested (Leopold and Logothetis, 1998).

Whatever its origin, functionally, the enhancement in early visual areas that occurs after microsaccades could act to regularize and synchronize sensory processing (Leopold and Logothetis, 1998), and this could also potentially help in image-stabilizing oculomotor reflexes. For example, very short-latency ocular following reflexes, which stabilize retinal images in the face of full-field image motion (Miles et al., 1986; Miles, 1997), are significantly enhanced after microsaccades, and with a time course similar to how early sensory areas in the visual system are sensitized (Chen and Hafed, 2013). This idea is similar to the idea that the excitability of early sensory processing is enhanced after large saccades (Rajkai et al., 2008).

At the level of *perception*, however, saccades and microsaccades cause changes in sensitivity that are very different from the post-movement enhancement that is seen in early visual areas. Specifically, it has been long known that visual sensitivity at the behavioral level is *reduced*, rather than enhanced, after saccades (Zuber and Stark, 1966; Diamond et al., 2000) and microsaccades (Zuber and Stark, 1966; Hafed and Krauzlis, 2010; Chen et al., 2015; Tian and Chen, 2015). The time course of such saccadic *suppression* is such that visual sensitivity: (1) is reduced even before the eye begins to move (but see next section for a small caveat); (2) is maximal during the eye movement itself; and (3) stays reduced for almost ~100 ms after the eye movement has ended (i.e., when early visual areas might be experiencing enhanced firing rates). Even though some early visual areas, like LGN, do also show suppression of activity during microsaccades and before the post-movement enhancement alluded to above (Leopold and Logothetis, 1998; Martinez-Conde et al., 2002; Kagan et al., 2008; Bosman et al., 2009; Herrington et al., 2009; Martinez-Conde et al., 2013), their whole time course of modulation relative to movement onset (i.e., before, during, and after the microsaccade) is distinct from the time course of the perceptual phenomenon of saccadic or microsaccadic suppression (for a summary of microsaccadic effects in different brain areas, see Figure 4 of Martinez-Conde et al., 2013). While it is certainly conceivable that perception may be related to these modulations of early visual areas in a complex manner (as opposed to a simple 1:1 correlation with firing rates), the disparity between perception and simple firing rates in early visual areas has prompted a search for possible brain areas that may more directly correlate with (at the level of firing rates), and potentially mediate, the perceptual phenomenon of microsaccadic suppression.

**Figure 2:**
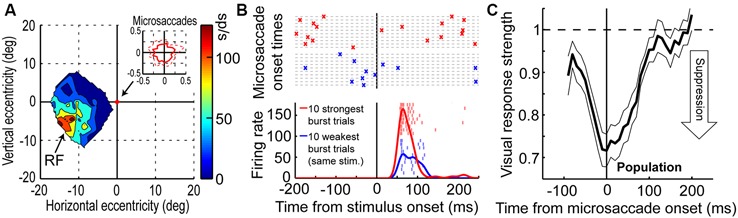
**Microsaccadic suppression of visual bursts in the primate SC. (A)** Visual RF map of a sample SC neuron from the study of Hafed and Krauzlis (2010). The neuron exhibited robust responses to locations in the lower-left quadrant of the visual field, with eccentricities >10 degrees. The inset shows the extent of microsaccade amplitudes that the monkey generated during the session. **(B)** Responses of the neuron in **(A)** to a bright stimulus placed at the best RF location. The bottom panel shows the firing rate of the neuron to the exact same stimulus for the ten trials with the weakest (blue) or strongest (red) visual bursts. Individual tick marks are individual action potentials from individual trials. As can be seen, there was a large dynamic range of neuronal response to the exact same stimulus. The top panel shows the times of microsaccades (as crosses) on the same ten weak (blue) or ten strong (red) burst trials. As can be seen, visual bursts were strongly suppressed whenever microsaccades occurred near stimulus onset (blue). **(C)** Normalized visual burst strength in the population of SC visual-motor neurons from the study of Hafed and Krauzlis (2010) as a function of when a stimulus appeared relative to the onset of a microsaccade. A suppression profile similar to behavioral saccadic suppression profiles can be observed. This figure was adapted with permission from Hafed and Krauzlis (2010).

Visual-motor neurons of the SC have turned out to more closely correlate with the perceptual phenomenon of microsaccadic suppression than early visual areas (Hafed and Krauzlis, 2010), at least based on simple firing rates. Figures 2A,B demonstrate this for a sample SC visual-motor neuron, whose visual (and motor) RF was more eccentric than 10 degrees from the fovea (Figure 2A). This neuron was therefore not involved in microsaccade generation *per se* (i.e., it was not like the neuron of Figure 1A), but it was instead tuned for visual locations or saccade endpoints in the periphery. While the monkey fixated, Hafed and Krauzlis presented a bright stimulus at the best location expected to elicit visual responses by this neuron (i.e., >10 degrees from the fovea). The scale of microsaccade amplitudes that the monkey generated is shown in the inset of Figure 2A, and it was more than two orders of magnitude smaller than the eccentricity of the stimulus, and also significantly smaller in size than the RF size itself. Thus, the eye movements were never directed towards the RF stimulus location, and they also caused minimal movement of the stimulus location relative to the center of the RF. Despite that, neuronal sensitivity to the same stimulus was dramatically altered around microsaccades (Figure 2B); the weakest visual bursts occurred on trials in which the monkey happened to generate a microsaccade in close temporal proximity to stimulus onset (Hafed and Krauzlis, 2010).

The time course of microsaccadic suppression seen in Hafed and Krauzlis (2010) closely matches the perceptual phenomenon of microsaccadic or saccadic suppression (Zuber and Stark, 1966; Diamond et al., 2000; Johns et al., 2009; Hafed and Krauzlis, 2010; Ibbotson and Krekelberg, 2011). Figure 2C demonstrates this for the population of visual-motor SC neurons recorded by Hafed and Krauzlis (2010). Visual responses are significantly suppressed even before microsaccade onset (by up to ~100 ms), the suppression is maximal near movement onset, and the suppression persists even after microsaccades have ended (for up to ~100 ms). This last observation is distinct from early sensory areas, which show post-movement enhancement, but it is consistent with perceptual suppression effects (Ibbotson and Krekelberg, 2011). It is interesting to note how the time course of microsaccadic suppression in the SC (Figure 2C) is very similar to the time course of the movement-related command (Hafed and Krauzlis, 2012) contributing to microsaccade generation (the suppression time course is an inverse of the elevation of movement-related activity in Figure 1A). It is also important to note that the modulations in Figure 2 cannot be accounted for by translation of the visual stimulus over the RF of the recorded neurons by the eye movements. Because microsaccades are so small compared to the sizes of the RF’s studied in Hafed and Krauzlis (2010), the stimulus onsets always occurred near the neurons’ preferred RF locations, and the suppression effects are much bigger than those predicted by small RF image displacements.

Thus, it seems that visual-motor areas like the SC are the areas that may most closely match the perceptual phenomenon of microsaccadic suppression. More recently, peri-microsaccadic modulations in the frontal eye fields (FEF) that are similar to SC modulations have also been identified (Chen et al., 2015). Intuitively, the idea that visual-motor areas like the SC and FEF would more closely match the perceptual effects might make sense: activity in the SC and FEF is more concerned with the behavioral relevance or salience of a stimulus than with its specific visual attributes (Krauzlis et al., 2013; Squire et al., 2013), and this might be sufficient to modulate perception in the manner that saccadic suppression does. Perhaps more interestingly, this observation of visual-motor neurons being closely related to the perceptual effects suggests that a single neuron type (in this case, visual-motor saccade-related SC neurons) can potentially subserve both perception-related modulations as well as saccade generation: the neurons in Figure 2 are modulated in their visual bursts in a manner that reflects perceptual suppression (Hafed and Krauzlis, 2010), and they (i.e., the *same* neurons) would also be activated if the monkeys were to later generate saccades to the appearing stimuli. As will become more clear in the Section “Implications of Peri-Microsaccadic Changes in Vision” below, this might be considered to be an example of one interpretation of the classic pre-motor theory of attention (Rizzolatti et al., 1994; Sheliga et al., 1994; Kustov and Robinson, 1996), in which it was presumed that circuits for saccade generation may also subserve modulations in visual representations necessary for covert visual attention. In this case, not only would the SC (or FEF) be involved in both attention and saccade generation, but even within these structures, it may be the same types of neurons (e.g., SC visual-motor saccade-related neurons) that are recruited in both.

Having said that, we should note here that for large saccades, cortical areas (albeit higher in the visual hierarchy than V1) have also been suggested to be implicated in large saccadic suppression (Bremmer et al., 2009). Some of these areas exhibit post-saccadic enhancement as well as the suppression effect (like in V1; Snodderly et al., 2001; Kagan et al., 2008), but their suppression time course is nonetheless well-correlated with the perceptual phenomenon of saccadic suppression (Bremmer et al., 2009). While this study of large saccades has not explicitly tested for the effects of microsaccades (but see Herrington et al., 2009; for a subset of the areas covered in Bremmer et al., 2009), its results do suggest a possible role for higher cortical areas in microsaccadic suppression as well. We view this as still consistent with the view that early visual areas (e.g., LGN and V1) may not necessarily correlate with perceptual effects as much as higher areas that are more concerned with behavioral relevance or salience, especially when one considers simple firing rate modulations. This sentiment was also recently suggested in the context of the effects of large saccades and microsaccades on V1 activity (McFarland et al., 2015), in which the authors favored the possibility that there may be multiple pathways for perisaccadic effects, with some pathways (e.g., pulvinar to MT) being involved in perceptual suppression, but not necessarily other pathways (e.g., the pathway through LGN and V1).

## Pre-microsaccadic Enhancement

Almost all studies on the role of microsaccades in altering neuronal activity (e.g., Leopold and Logothetis, 1998; Martinez-Conde et al., 2000, 2002; Bosman et al., 2009; Herrington et al., 2009; Hafed and Krauzlis, 2010) have thus far primarily focused on the microsaccade as a distinct temporal event regardless of its kinematic properties. For example, a stimulus could be presented peri-microsaccadically, and changes in the neuronal representation of the stimulus would be subsequently analyzed (Hafed and Krauzlis, 2010). However, the direction of a microsaccade could matter a great deal for peri-microsaccadic changes, just like the landing endpoint of a large saccade would be expected to matter for peri-saccadic changes. For example, if one were to measure visual sensitivity at the endpoint of a large saccade, improved sensitivity might be observed before saccade execution, because of a strong coupling between attention and pre-saccadic processing (Rizzolatti et al., 1994; Sheliga et al., 1994; Deubel and Schneider, 1996; Kustov and Robinson, 1996; Rolfs and Carrasco, 2012). However, if pre-saccadic sensitivity was measured *opposite* the landing endpoint of a saccade, suppression of sensitivity might be expected (i.e., saccadic suppression), and this might be related to the phenomenon of perceptual stability in the face of disruptive eye movements (Diamond et al., 2000; Ross et al., 2001). In fact, even though this aspect of the data was not directly emphasized in the article itself, visual inspection of the results presented in Knöll et al. (2011) seems to suggest the existence of differential effects of sensitivity in the pre-saccadic interval for locations at the saccade landing point and opposite it; that is, visual inspection of their data suggests that sensitivity is higher for the upcoming saccade endpoint location (although this is purely based on visual inspection of their data figures, and without proper statistical analysis). For microsaccades, something similar could be envisioned, especially because at the level of the SC, different sets of neurons would be recruited to generate different sets of microsaccade amplitudes and directions (Hafed et al., 2009; Hafed and Krauzlis, 2012). Indeed, it was recently found in behavioral experiments in humans (Hafed, 2013) that suppression of peripheral visual sensitivity before the onset of a microsaccade (Hafed and Krauzlis, 2010) is not universal for all microsaccade directions. Rather, if one were to probe perceptual performance at a peripheral location *congruent* with the direction of an upcoming microsaccade, then strong improvements in perceptual performance, rather than reductions, can occur (Hafed, 2013; also see Figure 6C below). This suggests that the mere preparation to make a tiny microsaccade in one direction can have either enhanced or reduced perceptual performance depending on where a peripheral stimulus appears relative to the movement direction. We thus recently revisited the neurophysiological phenomenon of microsaccadic suppression, but now from the perspective of what happens in more detail during the pre-movement interval.

**Figure 3:**
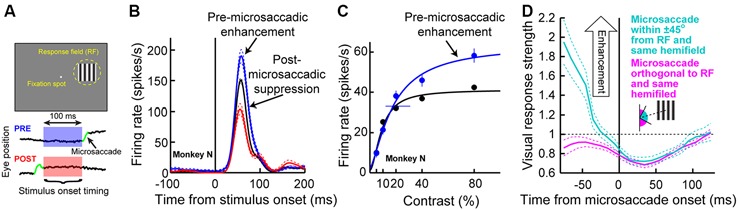
**Pre-microsaccadic enhancement of neuronal response gain. (A)** Experiment (Chen et al., 2015) in which a monkey simply fixated a small spot while a sine wave grating was presented inside an SC neuron’s RF. The bottom schematic shows the analysis logic: grating onset could either appear before (blue) or after (red) a microsaccade. **(B)** Visual response of a sample SC visual-motor neuron from the SC during the experiment of **(A)**. The black curve shows firing rate on trials in which the grating appeared without any microsaccades in the near temporal vicinity; a classic visual response was observed. If the grating appeared <100 ms before the onset of a microsaccade directed towards the RF location, the visual burst was strongly enhanced (blue). On the other hand, if the grating appeared after the same microsaccade, the visual burst was strongly suppressed (red). **(C)** The pre-microsaccadic enhancement of **(B)** acted a multiplicative gain modulation of the contrast sensitivity curve of the neuron when all sine wave grating contrasts were tested. **(D)** Time course of peri-microsaccadic modulation computed in a manner identical to that in Figure 2C, except that trials are now separated based on whether the stimulus was directed towards the stimulus location (light green) or away from that location but still within the same hemifield (magenta). As can be seen, pre-microsaccadic enhancement was specific to movements congruent with the RF location; pre-microsaccadic suppression was otherwise observed. After the microsaccade, suppression is equally strong for different movement directions. Thus, the net effect of peri-microsaccadic changes (when one considers both pre- and post-movement effects) is still differential based on different movement directions, because of the pre-movement difference. This figure was adapted with permission from Chen et al. (2015).

We recorded SC activity from pure visual and visual-motor neurons while monkeys fixated, and we presented a sine wave grating inside the neurons’ visual RF’s (Figure 3A; we also used spots of light in separate experiments in the same study, with similar results). We separated trials in analyses based on whether a stimulus appeared before or after a microsaccade (Figure 3A). We found that during the pre-movement interval, microsaccadic suppression (Hafed and Krauzlis, 2010) does not universally occur, which is consistent with the recent behavioral predictions of peri-microsaccadic influences on perception (Hafed, 2013). Instead, a direction-dependent *enhancement* of visual sensitivity occurs before microsaccade onset (Chen et al., 2015). Figure 3B demonstrates this effect for an example visual-motor SC neuron (Chen et al., 2015). The black curve shows the activity of the neuron if the stimulus (a high contrast sine wave grating) appears without microsaccades occurring near stimulus onset, and the red curve shows activity for the same stimulus but if the stimulus appears immediately after a microsaccade directed towards the RF location. Strong microsaccadic suppression is observed (compare red and black curves). However, if the same stimulus appears immediately *before* the same microsaccade (i.e., a microsaccade directed towards the RF location), strong response gain enhancement occurs (Figure 3B, blue curve). Thus, pre-microsaccadic enhancement, rather than suppression, can indeed occur, and it seems to scale neuronal contrast sensitivity curves in a multiplicative gain modulation manner (Chen et al., 2015; Figure 3C). Moreover, such enhancement also occurs for FEF neurons, which again show both pre-microsaccadic enhancement and post-microsaccadic suppression (Chen et al., 2015).

The results of Figures 3A–C might at first glance seem to be in contradiction with the time course of microsaccadic suppression shown in Figure 2C (Hafed and Krauzlis, 2010), as well as the time course of perceptual suppression in humans (Zuber and Stark, 1966). That is, Figure 2C as well as early behavioral evidence of microsaccadic suppression in humans (Zuber and Stark, 1966) both suggest the existence of *both* pre- and post-microsaccadic suppression. However, Figures 3A–C indicates pre-microsaccadic enhancement followed by post-microsaccadic suppression. It turns out that the difference lies in microsaccade directions. Plotting the time course of microsaccadic modulations from the study of Figure 3 in a similar manner to Figures 2C, 2C, 3D shows that if a microsaccade is not directed towards the RF location, robust pre-microsaccadic suppression does indeed occur (Chen et al., 2015), consistent with the earlier literature. Therefore, there appears to be a *selective gating* of neuronal response gain as a function of microsaccade direction: if the microsaccade is towards the RF location, pre-microsaccadic enhancement occurs; if it is not, pre-microsaccadic suppression occurs.

As mentioned above, such direction-dependent selective gating reflects perceptual effects seen in humans, which do depend on microsaccade direction (Hafed, 2013; also see Figure 6C below for more details). However, the mechanisms behind this observation are currently unknown. One possibility could be that this selective gating might reflect circuit wiring properties in the SC. For example, an excitatory pathway from the motor layers of this structure (i.e., the layers generating a saccade burst) to the sensory layers (i.e., the layers having pure visual bursts) exists (Ghitani et al., 2014). Interestingly, this excitation seems to spread laterally across eccentricities (Ghitani et al., 2014). Thus, a motor burst for a given saccade might be associated with excited visual bursts at different eccentricities that are dissociated from the movement endpoint. In the case of microsaccades, this would mean that neurons in the foveal portions of the motor layers of the SC (Figure 1A) would build up and burst to generate a microsaccade, while eccentric neurons in the visual layers might experience enhancement. Consistent with this idea, we also found robust pre-microsaccadic enhancement of visual bursts in the SC’s pure visual neurons (i.e., in the structure’s sensory layers), and not just in visual-motor neurons (Chen et al., 2015).

Other possibilities for the selective gating mechanism of Figure 3D, which need not be mutually exclusive from the excitatory circuit mechanism just mentioned, could include the idea that microsaccades and visual sensitivity could both be part of a rhythmic process in the brain (Gaarder et al., 1966; Hafed and Ignashchenkova, 2013), such that visual sensitivity waxes and wanes with a certain temporal structure that is related to the temporal structure of the saccadic system. Whatever the case may be, we anticipate that the mechanisms responsible for the results of Figure 3 will constitute an active area of research in the near future.

The modulation that we alluded to above of pure visual SC neurons in Chen et al. (2015) is particularly intriguing. Specifically, there seem to be important differences between visual and visual-motor neurons, and future investigations of these differences will advance our understanding of the mechanisms behind peri-saccadic phenomena in general, even for large saccades. For example, even though both visual and visual-motor SC neurons exhibit robust post-microsaccadic suppression (Chen et al., 2015), the suppression is significantly weaker in the visual neurons than in the visual-motor neurons (their Figures 2C,D and their Figures 3C,D). In particular, visual-motor neurons exhibit ~30% suppression (Hafed and Krauzlis, 2010; Chen et al., 2015), whereas visual neurons experience only ~15% suppression (Chen et al., 2015). Moreover, the time course and direction-dependance of peri-microsaccadic modulations of visual SC neurons are slightly different from those of SC visual-motor neurons (Chen et al., 2015), with the latter appearing to be more in line with perceptual alterations around microsaccades (Hafed, 2013). Both of these observations combined (i.e., weaker suppression in visual than visual-motor neurons, as well as weaker correlation with perceptual effects when one considers simple firing rate magnitudes) call for a recasting of hypothesized saccadic suppression phenomena, in which it was suggested that saccadic suppression (for large saccades) might originate, at least in part, through a suppressive circuit starting in the deeper layers of the SC (where visual-motor neurons are located) and inhibiting the superficial layers (where visual neurons are located; Phongphanphanee et al., 2011). Instead, what seems to happen is that the suppression is already implemented very robustly and strongly in the visual-motor neurons themselves. This is further evidence that a single type of neuron (e.g., visual-motor SC neurons) can potentially mediate both perceptual effects as well as saccade generation, consistent with one interpretation of the pre-motor theory of attention alluded to above. Of course, this does not preclude that other brain areas may also be involved in peri-microsaccadic phenomena, as in the case of large saccades and cortical modulations (Bremmer et al., 2009). However, it does indicate that, at least, within the SC, it is visual-motor neurons that may be important for a variety of phenomena related to not only peri-microsaccadic changes (as in this review), but also attention (Zénon and Krauzlis, 2012) and target selection (Krauzlis and Carello, 2003).

Finally, the amount of post-microsaccadic suppression in Figure 3D is similar for different microsaccade directions relative to the RF location, unlike the pre-movement modulations. This means that studies measuring perception immediately after the end of a microsaccade would in reality be measuring dramatically altered visual representations from those occurring without nearby movements (Tian and Chen, 2015), and that this effect would not strongly depend on the direction of the microsaccade. Thus, there are differential direction-dependent effects before microsaccades and general suppression later (Figure 3), supporting the view that peri-microsaccadic modulations in vision are decidedly more *sophisticated* than global suppression or global retinal-image refreshing effects. This in turn means that there can be important differential effects of microsaccades that can have a significant impact on the interpretation of experiments enforcing prolonged fixation, as we explain in more detail in the Section “Implications of Peri-Microsaccadic Changes in Vision”.

## Peri-Microsaccadic Changes in the Representation of Space

Besides saccadic suppression, large saccades are also associated with momentary distortions in the perception of space. Brief flashes of light, which are bright enough to overcome the effects of saccadic suppression and are therefore nonetheless perceived, are consistently and reliably mislocalized (Matin and Pearce, 1965; Matin et al., 1969, 1970; O’Regan, 1984; Honda, 1989, 1991; Cai et al., 1997; Ross et al., 1997; Lappe et al., 2000; Pola, 2011). Subjects who try these types of experiments can have very high confidence in their perceptual localization reports, but the reports themselves are still often very erroneous. There are disparate theories about the origins of these peri-saccadic mislocalization effects (Ross et al., 2001; Zimmermann et al., 2014), and the patterns of mislocalization themselves can vary widely according to the experimental condition (Lappe et al., 2000; Pola, 2011). For example, under some conditions, a brief flash presented peri-saccadically would be misperceived as being shifted in the direction of the saccade (Honda, 1989, 1991), whereas under other conditions, the pattern of perceptual shifts critically depends on the location of the brief flash relative to the saccade endpoint (Ross et al., 1997): if a flash is presented farther away from the saccade endpoint relative to current fixation, the flash is misperceived as being shifted backwards towards fixation and opposite the saccade direction; if it is presented nearer to fixation from the saccade endpoint, it is perceived erroneously as being shifted away from fixation in the same direction as the saccade. In other words, it is as if space is “compressed” towards the saccade endpoint (Ross et al., 1997). This kind of perceptual distortion (i.e., an apparent compression of space) is the one that is most reliably observed when ambient illumination provides the visual system with reliable visual references (Lappe et al., 2000).

Neurophysiologically, spatial representations are also altered in complex ways during peri-saccadic intervals, although how these alterations account for perceptual mislocalization effects remains to be understood. For example, visual RF’s in the lateral intraparietal area (LIP), FEF, and other visual areas can exhibit remarkable malleability around the time of large saccades (Duhamel et al., 1992; Walker et al., 1995; Umeno and Goldberg, 1997; Nakamura and Colby, 2002; Sommer and Wurtz, 2006). The first evidence demonstrating this came from studies in which the RF’s of LIP neurons appeared to “remap” in an anticipatory manner to the locations that they would occupy retinotopically after the eye completes its saccade (Duhamel et al., 1992): that is, a neuron that responds to one retinotopic location would momentarily now respond to a location outside its classic RF boundary, but a location that is still consistent with the retinotopic RF location if the eye lands correctly on the intended saccade target. This phenomenon has been replicated in several areas including the FEF (Umeno and Goldberg, 1997; Sommer and Wurtz, 2006), V3 (Nakamura and Colby, 2002), and SC (Walker et al., 1995). More recently, a slightly different pattern of RF shifts was also discovered. Specifically, neurons in V4 (Tolias et al., 2001) and FEF (Zirnsak et al., 2014) appear to shift the position of their RF’s peri-saccadically, but this time in a manner consistent with a convergence of neuronal resources (i.e., RF’s) towards the intended saccade endpoint. Some models of peri-saccadic RF shifts posit that such convergence of neuronal RF’s towards the saccade endpoint can arise through an interaction between pre-motor activity in saccade generation maps and stimulus-induced visual bursts in retinotopic visual maps (Hamker et al., 2008; Zirnsak et al., 2014), and such models even show that both “remapping” RF shifts as well as “convergence” RF shifts can be observed with similar mechanisms (Zirnsak et al., 2010). Even more recently, an important role for eye position signals in accounting for some perceptual mislocalization effects has also been discovered (Morris et al., 2012, 2013).

**Figure 4:**
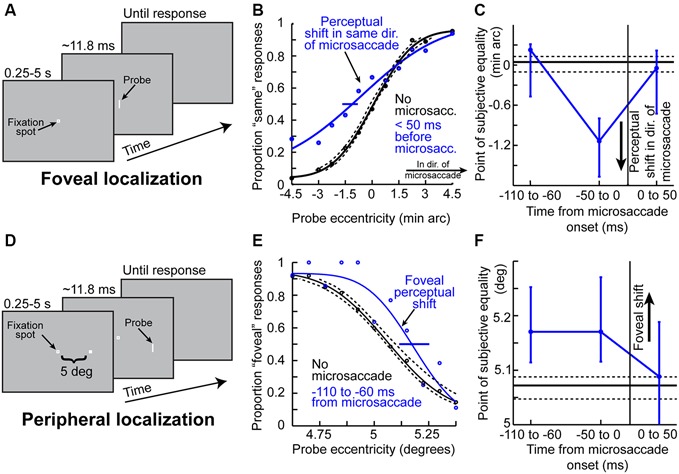
**Peri-microsaccadic mislocalization of briefly flashed stimuli. (A)** Foveal localization task of Hafed (2013). Human subjects fixated, and a brief flash was presented at a variable horizontal displacement from screen center. **(B)** Perceptual localization performance in the task of **(A)** when the brief flashed probe was presented without microsaccades (black) or immediately before the onset of a microsaccade (blue). In baseline (black), the percept was veridical (the psychometric curve’s point of subjective equality was at the point in which the probe was ambiguous in location). However, when the probe was presented before a microsaccade, the point of subjective equality was shifted such that subjects misperceived the stimulus as being shifted in the direction of the upcoming microsaccade. **(C)** The mislocalization in **(B)** was time-locked to microsaccade onset, with a similar time course to pre-microsaccadic modulations seen in Figures 2, 3. **(D)** Similar localization task but performed in the periphery (Hafed, 2013). Subjects had to judge the location of a brief probe relative to a peripheral reference. **(E)** Mislocalization of the brief probe also occurred in the pre-microsaccadic interval, but it was now directed foveally opposite the direction of the microsaccade. Thus, the results of foveal and peripheral localization together suggest that peri-microsaccadic mislocalization is a correlate of large saccadic compression. **(F)** The time course of peripheral mislocalization was also synchronized with microsaccade onset, as in **(C)**. This figure was adapted with permission from Hafed (2013).

Given the results of Figures 1–3 so far, it might be asked whether microsaccades might also be associated with peri-movement distortions in the perception of space. Hafed (2013) has demonstrated this phenomenon using behavioral experiments in humans. The experiments he used were conceptually similar to experiments of peri-saccadic mislocalization (Ross et al., 1997). Specifically, Hafed (2013) has tested for a microsaccadic correlate of saccadic compression. To do so, localization of brief flashed probes was tested either foveally (Figure 4A) or peripherally (Figure 4D). Hafed (2013) found that if a foveal probe appears <50 ms before the onset of a microsaccade, its location is consistently misperceived by a small amount in the direction of the upcoming movement (Figure 4B). This perceptual mislocalization is time-locked (Figure 4C) to microsaccade onset and peaks in the pre-movement interval, which is analogous to the results of Figure 3, and also similar to large saccadic compression effects (Ross et al., 1997, 2001). With peripheral localization (Figure 4D), it was found that peri-microsaccadic mislocalization does still take place but now in the opposite direction from the microsaccade (i.e., back towards the foveal endpoint of the movement). Once again, the mislocalization is time-locked to microsaccade onset (Figure 4F), but it extends further back in time than foveal localization. Interestingly, this time course (Figure 4F) is similar to how pre-microsaccadic enhancement of peripheral SC visual-motor neurons also extends further back in time (Figure 3D).

Therefore, microsaccades are associated with an analog of “saccadic compression”. From a theoretical perspective, the mechanisms of this microsaccadic compression seem to be in line with models of large saccadic compression, in which the distribution of motor activity associated with microsaccade generation (Hafed et al., 2009) interacts with retinotopic visual responses to the probes. In fact, Hafed (2013) has simulated the phenomenon with a model that had been used earlier to simulate large saccadic compression (Hamker et al., 2008), but now taking into account the distribution of SC activity during fixation (Hafed and Krauzlis, 2008) and microsaccades (Hafed and Krauzlis, 2012).

Of course, it should be noted that the mislocalization effects seen around the time of microsaccades (Figure 4) are small in magnitude, but this is to be expected given the very small sizes of the eye movements. Nonetheless, it is remarkable that a movement less than a quarter or a fifth of a degree in amplitude can influence perception in a measurable manner at a much larger eccentricity. It should also be noted that there currently exist no neurophysiological studies exploring the possible mechanisms for such mislocalization. For example, there are currently no published results on potential RF shifts around the time of microsaccades, analogous to the peri-saccadic shifts of RF’s (whether “remapping” or “convergence” shifts) that we described above. This is a frontier of research on the neuronal mechanisms for peri-microsaccadic changes in visual perception.

## Far-Reaching Effects of Microsaccades

As we mentioned above, the fact that microsaccades are small does not in any way imply that microsaccades cannot have a strong impact. In fact, a potential “extra-retinal” impact of microsaccades can be much stronger than a retinal impact. For example, in the periphery where RF’s are large, a tiny image shift caused by microsaccades might be too small to strongly alter the visual representation of a stimulus positioned over the RF. However, if there was an active, extra-retinal signal associated with the motor generation of microsaccades, and if this signal were to influence visual representations, then this signal might affect neuronal responses independent of the size of the movement being generated, or the eccentricity and size of the RF being stimulated.

**Figure 5:**
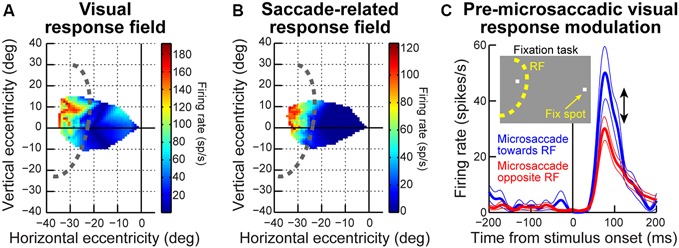
**Far-reaching effects of peri-microsaccadic modulations. (A)** Visual RF map of a sample SC neuron from the data set of Chen et al. (2015). The neuron exhibited robust responses to locations in the upper-left quadrant of the visual field, with eccentricities >30 degrees. Note how the neuron was so eccentric that we could not map its entire extent given our display system. The dashed curve indicates our estimate of the size and shape of the neuron’s RF. As can be seen, the neuron had an RF center that was likely >40 degrees in eccentricity (i.e., near the end of the oculomotor range). **(B)** Same as the RF map in **(A)** for the same neuron, but now measuring pre-saccadic firing rates, to indicate the *motor RF* of the neuron. The neuron did not burst for any microsaccades, and was instead tuned for large saccades >~30 degrees in amplitude. Thus, any modulation in the neuron’s activity around microsaccades cannot be explained by a microsaccade-related motor discharge. **(C)** Nonetheless, the neuron was dramatically modulated by tiny microsaccades. In this panel, we show visual bursts of this neuron when a stimulus was presented at the shown inset location (i.e., very far from fixation but still inside the RF of the neuron). The logic of this experiment was similar to that of Figures 2, 3. As can be seen, visual burst strength was very different on trials in which the stimulus appeared before a microsaccade towards the RF location (blue) and on trials in which the same stimulus appeared before a microsaccade opposite the RF location (red). This happened even though the stimulus was placed at an eccentricity much larger than the microsaccades, and with a neuron with a very large RF size. A potential retinal-image shift of the stimulus by tiny microsaccades is insufficient to account for such a large change in the dynamic range of the neuronal response. Thus, the effects of Figures 2, 3 can be very far-reaching in space.

Even though the exact mechanisms are not yet fully known, all of the studies summarized above (Figures 1–4) strongly support the idea that microsaccades can have a strong impact even in the far periphery. For example, the sample neuron of Figure 2A is affected by microsaccades even though it is tuned for much larger eccentricities. Similarly, the magnitudes of pre-microsaccadic enhancement and post-microsaccadic suppression summarized in Figure 3D are substantial (Chen et al., 2015). To further demonstrate this idea in an even more compelling manner, we show in Figure 5 a sample SC visual-motor neuron recorded as part of the data set of Chen et al. (2015). The neuron’s visual and saccade-related RF’s are shown in Figures 5A,B respectively, demonstrating that the neuron was extremely peripheral (extending beyond our stimulus display limits and preferring eccentricities at least >40 degrees). As described in Chen et al. (2015), we measured visual burst strength when a stimulus (inset in Figure 5C) appeared before a microsaccade either towards (blue curve in Figure 5C) or opposite (red curve in Figure 5C) the RF location. There was a major difference in the response gain of the neuron for the same stimulus (Chen et al., 2015). Thus, a microsaccade less than a quarter or a fifth of a degree in amplitude can have a substantial impact on a neuron that is tuned for a location >40 degrees in eccentricity. This intriguing far-reaching impact is also very strongly direction-dependent, with an almost halving of the response gain of the neuron when microsaccade direction becomes opposite the RF location compared to being towards it (Figure 5C). Thus, the results of Chen et al. (2015) extend to quite far eccentricities, near the limit of the oculomotor range.

Besides far-reaching effects in space, microsaccades can also have a far-reaching effect in time. For example, the peri-microsaccadic response gain changes summarized in Figures 2, 3 above extend for a period of ~200 ms centered around the time of microsaccade onset. Moreover, Chen et al. (2015) have found that for neurons with a sustained visual response, response gain enhancement also extends to the portion of the response that is sustained. That is, not only is the visual burst enhanced for a stimulus appearing before microsaccades, but the sustained response (that persists as long as the stimulus is presented) is also elevated compared to when no microsaccades occur. This finding is particularly intriguing, especially because both the onset event and the microsaccade itself had long ended.

Therefore, peri-microsaccadic changes in vision occur in extended periods of time and for very large portions of space. We next describe how these two observations can strongly matter for the interpretation of a variety of experiments involving gaze fixation.

## Implications of Peri-Microsaccadic Changes in Vision

Peri-saccadic changes in vision for large saccades are often thought of from the perspective of perceptual stability mechanisms. In other words, saccades cause massive disruption in the flow of visual information from the eyes to the rest of the brain, and peri-saccadic phenomena observed in the laboratory may reflect the existence of retinal and extra-retinal mechanisms used by the visual system in order to stabilize percepts in the face of such disruption (Ross et al., 2001). We believe that peri-microsaccadic changes in vision may also be looked upon from this perspective. However, in the case of microsaccades, an additional perspective that needs to be considered emerges, and this has to do with the conditions under which these small eye movements occur. In every experiment requiring prolonged gaze fixation, microsaccades will inescapably be present, with varying degrees of frequency of occurrence. If each microsaccade command (Figure 1; Hafed et al., 2009; Hafed and Krauzlis, 2012) is accompanied by peri-movement changes like the ones summarized above (Figures 2–5), then it would be expected that vision during such experiments will be repeatedly modulated whenever microsaccades occur. Moreover, the modulations in vision that do happen will often be identical in form to the modulations that some of these experiments aim to uncover in the first place (Chen et al., 2015). Thus, peri-microsaccadic modulations in vision would constitute at least a partial component of the mechanisms originally being investigated by these experiments.

**Figure 6:**
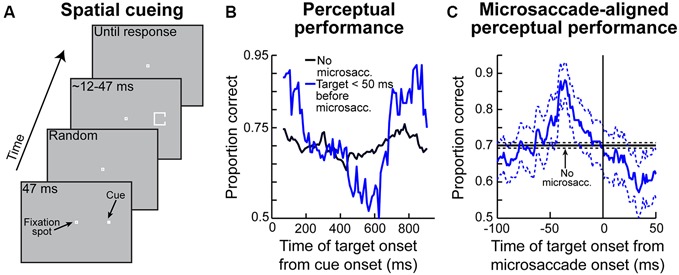
**Contributions of peri-microsaccadic changes in vision to perceptual performance in attentional tasks. (A)** Classic attentional cueing task in which spatial attentional allocation is instructed by a cue, and the effects of attentional cueing are probed with a perceptual discriminandum presented some time after cue onset. This task was the one used in Hafed (2013). **(B)** Perceptual performance in the task of **(A)** as a function of when the perceptual target appeared after cue onset. The black curve shows performance on trials in which the target appeared without nearby microsaccades (Hafed, 2013). The blue curve shows performance in the same task and for the same stimulus when the target appeared in the critical pre-microsaccadic interval of Figures 3D, 3D, 4. As can be seen, there were massive perceptual oscillations in performance in the pre-microsaccadic interval. **(C)** These oscillations could be understood when perceptual performance was plotted as a function of when the peripheral target appeared relative to the onset of a microsaccade directed towards its location. In this case, strong perceptual enhancement was observed if the peripheral target appeared in the pre-movement interval in which pre-microsaccadic enhancement is seen neuronally (Figure 3D). When the microsaccade was opposite the target, the enhancement was not present (Hafed, 2013). This explains the massive oscillations in **(B)**. That is, microsaccades were predominantly towards the target location early after cue onset and much later after cue onset, but they were opposite the target location in between (i.e., the microsaccades were oscillating in direction; Hafed, 2013). Thus, there was pre-microsaccadic enhancement in performance early and late after cue onset but pre-microsaccadic suppression in between. This figure was adapted with permission from Hafed (2013).

Recent results on the relationships between microsaccades and covert visual attention provide the clearest example of the above sentiment to date. Early in this century, it was discovered that during spatial attentional cueing experiments (e.g., Figure 6A), the patterns of microsaccades that occur in these experiments are far from random (Hafed and Clark, 2002; Engbert and Kliegl, 2003; Hafed et al., 2011). Microsaccade directions are biased in a dynamic fashion by cue location, first being predominantly shifted in the direction of an abruptly appearing cue and then oscillating back to being biased opposite the cue. This phenomenon has been replicated several times, and it was initially interpreted as meaning that microsaccades provide a probabilistic “read-out” of the state of covert attention after cue onset. However, it has now become clear that these modulations in microsaccades are, in reality, automatic and reflexive modulations, occurring even in the absence of any attentional task requirements (Rolfs et al., 2008; Hafed and Ignashchenkova, 2013). These modulations may be thought of as being similar to how large saccades reflexively react to even the briefest and most irrelevant of flashes in the phenomenon known as “saccadic inhibition” (Reingold and Stampe, 2002). Thus, modulations in microsaccades after cue onset reflect a sensory-transient-driven “resetting” of the (rhythmic) saccadic system (Hafed and Ignashchenkova, 2013): saccades are repetitively generated in a continuous, rhythmic manner even during fixation, and any stimulus onset (like cue onsets) simply resets this rhythm (Hafed and Ignashchenkova, 2013). This means that during spatial cueing experiments (e.g., Figure 6A), microsaccades would not only happen in these experiments (Hafed and Clark, 2002; Engbert and Kliegl, 2003), but they would also be modulated systematically. This idea of a reflexive modulation of microsaccades by sensory transients is further supported by the observation that when monkeys perform tens of thousands of trials of a given cueing task during training, the modulations of microsaccades that they exhibit are highly stereotypical and repeatable, without signs of adaptation due to extensive exposure to the task or stimuli (Hafed et al., 2011, 2013; Hafed and Ignashchenkova, 2013).

Given the above, if one were to now consider that each microsaccade occurring in spatial cueing tasks is associated with peri-movement changes in vision, then one should expect to observe very specific modulations in attentional performance during these cueing tasks. This is exactly what Hafed (2013) found. This author has hypothesized that target onset after an attentional cue not only comes at different times relative to cue onset (which is dictated by the experimental design; Figure 6A), but it also comes at different times relative to microsaccades. He therefore measured perceptual performance in the discrimination of the target onset (which is the behavioral measure of attentional allocation in these tasks) based on when the target appears relative to microsaccades. Hafed (2013) found that modulations in attentional performance after cue onset are very strongly magnified if the target is presented within <50 ms before the onset of a microsaccade compared to when it appears without microsaccades (Figure 6B). Interestingly, this pre-microsaccadic interval of magnified attentional effects is essentially the same interval in which this author had earlier found alterations in spatial perception in his subjects during an unrelated localization task (Figures 4C,F). In fact, replotting the data of Figure 6B but now as a function of the time of peripheral target onset relative to microsaccade onset rather than relative to cue onset, it can be seen that peripheral attentional performance in this spatial cueing task exhibits clear peri-microsaccadic modulations (Figure 6C): during the same interval before microsaccade onset as that in which spatial perceptual alteration occurs (Figures 4C,F), strong modulation in target-driven response also takes place (Figure 6C).

Note that Figure 6C shows target-related modulations for the peripheral target appearing in the *same* direction as the upcoming microsaccade. If the peripheral target appears opposite the microsaccade, then suppression of performance occurs (Hafed, 2013), consistent with the neurophysiological results of Figure 3. This direction-dependance explains the strong oscillation in performance in the blue curve of Figure 6B—because microsaccades oscillate in direction relative to the cue location, they are initially in the direction of the target immediately after cue onset (explaining the massive rise in Figure 6B early after cue onset), but they flip to being opposite the target (explaining the dip to near chance performance) before flipping back to being biased towards the target again (explaining the final rise in performance in Figure 6B; Hafed, 2013). Therefore, the direction-specificity of peri-microsaccadic modulations seen in Figures 3–4 has a direct correlate in the modulations in attentional performance in Figure 6B.

Also note that if the target appears after microsaccades in Figure 6C, suppression of performance occurs, consistent with post-microsaccadic suppression (Figures 2–3; Hafed and Krauzlis, 2010; Chen et al., 2015). Finally, note that the peri-microsaccadic modulations of attentional performance seen in Figure 6 are very large in magnitude compared to normal changes in attentional performance using classic analysis techniques in these types of tasks. Thus, if microsaccades were to occur on even a small minority of trials with the target appearing in the critical peri-microsaccadic interval, then a contribution of microsaccades to overall behavioral performance can still be observed. This is also consistent with the large effects of microsaccades seen neuronally (Figures 2, 3, 5).

Taken together, the results of Figure 6 suggest that the links between microsaccades and covert attentional shifts might be stronger than general correlations, or the idea that microsaccades are a probabilistic read-out of the instantaneous state of covert attention. Instead, there is almost a deterministic link between microsaccades and covert attention, in the sense that attentional performance is altered before the execution of individual microsaccades. If that is the case, then one might expect to observe *neuronal* *signatures* of covert visual attention before microsaccades as well, even when monkeys do not perform any attentional task at all. This is what was found (Chen et al., 2015). Besides response gain enhancement (Figure 3), which is one of the most classic signatures of attentional allocation (Moran and Desimone, 1985), other effects that were reported in the study of Chen and colleagues point to a strong link between microsaccades and covert attentional signatures. For example, multiplicative gain modulation of contrast sensitivity curves seems to occur (Figure 3C), consistent with the effects of attention (Reynolds and Heeger, 2009). Moreover, neuronal response gain enhancement is accompanied by improved signal-to-noise ratio, and it also persists as an elevation of sustained visual activity for neurons exhibiting sustained responses to stimuli presented over their RF’s (Chen et al., 2015). Both of these effects are established effects associated with attentional allocation (Roelfsema et al., 1998; Mitchell et al., 2007). Therefore, peri-microsaccadic changes in neuronal activity encompass a wide range of phenomena that are also observed in experiments on covert visual attention (Morris, 2015).

**Figure 7:**
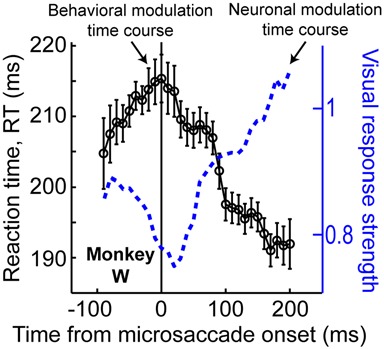
**Contributions of peri-microsaccadic changes in vision during speeded detection tasks.** If monkeys are asked to detect an appearing stimulus as fast as possible, their reaction time (RT) is modulated if a stimulus occurs near microsaccade onset (Hafed and Krauzlis, 2010). Remarkably, the pattern of RT modulations is correlated almost perfectly with the pattern of neuronal modulations of *visual bursts* in the SC (see the blue neuronal modulation curve, which is similar to Figure 2C but for only the monkey whose behavioral data is shown in the present figure). Specifically, the stronger the visual burst, the faster the RT, and *vice versa*. Thus, in any experiment in which visual bursts are affected, predictable changes in RT can also be directly expected. This result, coupled with the predictable changes in visual bursts in Figure 3D, suggests that peri-microsaccadic changes in vision can play a substantial role in experiments in which behavioral detection performance is experimentally probed (see Figure 8). This figure was adapted with permission from Hafed and Krauzlis (2010).

The results of Figure 6, coupled with those of Figure 3, also suggest that peri-microsaccadic modulations of vision can potentially contribute to interpretations of other classic attentional phenomena. For example, Hafed and Krauzlis (2010) have shown that microsaccades influence reaction time (RT) in a manner that is directly related to how they affect visual bursts, and also similarly to how large saccades might alter RT (Johns et al., 2009). Specifically, Hafed and Krauzlis instructed monkeys to detect a stimulus as soon as it appears by making a saccade towards it. They found that detection RT is strongly influenced by microsaccades near stimulus onset, and that the pattern of RT effects is almost entirely explained by the peri-microsaccadic modulations in SC visual burst strength (Figure 7): the weaker the visual burst, the later the RT; and *vice versa* for strong visual bursts. Because visual bursts are a significant determinant of saccadic RT (Boehnke and Munoz, 2008; Marino et al., 2012), this result is consistent with the idea that microsaccadic modulations of visual bursts can have strong and direct consequences on RT behavior. If visual bursts are enhanced around some microsaccades (e.g., Figure 3), then RT’s should be faster, and if they are suppressed around other microsaccades (e.g., Figures 2, 3), then RT’s should be slower. More importantly, since *manual* button press RT’s are also directly correlated with the properties of early visual responses in the visual system (Breitmeyer, 1975), one could naturally also anticipate that peri-microsaccadic modulations in visual burst strength (Figures 2, 3, 7) would also directly influence *manual* button press RT’s. This leads to an extremely intriguing *hypothesis* about the classic Posner attentional cueing task (Posner, 1980; Posner and Cohen, 1984; Posner et al., 1985). This task, which has been used to great effect in advancing our understanding of covert visual attention, is conceptually identical to that in Figure 6A, except that subjects are instructed to detect target onset by saccadic or manual responses (with RT being the behavioral measure of attention). In this task, saccadic or manual RT’s are modulated in a very systematic manner, such that RT’s are faster at cued vs. uncued locations soon after cue onset (a phenomenon called “attentional capture”), but they are slower at cued vs. uncued locations later (a phenomenon called “inhibition of return” or “IOR”; Posner, 1980; Posner and Cohen, 1984; Posner et al., 1985). Moreover, neuronal recordings from the SC during Posner cueing have shown that visual bursts after target onset are indeed stronger when the target appears soon after cue onset (i.e., for attentional capture) and weaker when the target appears later (i.e., for IOR; Dorris et al., 2002; Fecteau et al., 2004; Fecteau and Munoz, 2005). However, we now know that in Posner cueing, microsaccades are modulated in an automatic and highly systematic manner (Hafed and Ignashchenkova, 2013): early after cue onset, the microsaccades are primarily directed towards the cued location, and later, they are directed away from the cued location. Thus, if a target appears at the cued location with short latencies from cue onset, then it could benefit from enhanced visual bursts (Figure 3D) because of congruency of its location with microsaccades (i.e., pre-microsaccadic enhancement). Alternatively, if the same target appears later after cue onset, then microsaccades will have flipped back to being opposite the cue. Thus, the target-related visual burst could be suppressed (Figure 3D), and RT’s will be longer (i.e., pre-microsaccadic suppression). Thus, peri-microsaccadic modulations of vision might be expected to contribute to Posner cueing effects.

**Figure 8:**
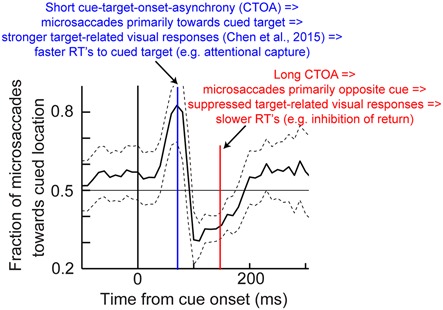
**Hypothesis on how peri-microsaccadic influences on speeded detection performance can potentially account for Posner cueing effects.** In Posner cueing tasks, an attentional cue is presented followed by a target, similar to Figure 6A. It is now known that cue onset reflexively resets microsaccadic rhythms (Hafed and Ignashchenkova, 2013), such that microsaccades are towards the cue shortly after its onset, but opposite the cue later. We hypothesize that if a target appears at different times after cue onset (e.g., blue or red), then the target will be appearing either in pre-microsaccadic intervals in which response gain enhancement occurs (Figure 3D) or in similar intervals in which response gain suppression occurs (Figure 3D; depending on microsaccade direction). Thus, a model in which *only* peri-microsaccadic modulations of visual bursts like those in Figure 3D influence RT like in Figure 7 can be *sufficient* to account for Posner cueing effects. This theoretical hypothesis does not in any way deny the “concept” of attention in general; it merely indicates that microsaccades can be a critical component of the mechanisms of covert visual attention, and this is consistent with several lines of evidence highlighted in this review. The microsaccade direction data shown in this figure have been adapted with permission from Hafed and Ignashchenkova (2013).

Thus, an alternative interpretation of Posner cueing effects, which is distinct from the classic attentional interpretation, posits that if target onset appears at a phase in which microsaccades are towards its location (regardless of prior cue location), then “attentional capture” might be observed because of pre-microsaccadic enhancement of visual bursts (Figure 3D). On the other hand, if target onset comes when microsaccades are biased in the opposite direction from its location, then suppressed visual bursts would be expected (Figure 3D); IOR might therefore be observed. This hypothesis, the logic of which is shown in Figure 8, suggests that peri-microsaccadic changes in target-related visual activity in Posner cueing *may be sufficient* to replicate both attentional capture and IOR. If this is true, then it would represent an intriguingly different perspective on Posner cueing effects, especially because according to this view, the location of the prior cue is somewhat irrelevant to the modulations in behavioral performance; performance is simply dictated by peri-microsaccadic changes in neuronal visual bursts (and therefore RT), and the role of the cue is to simply reset (like any other sensory transient) the ongoing microsaccadic rhythm of the oculomotor system. While this hypothesis remains to be demonstrated, we have unpublished evidence from computational modeling and experiments supporting it (unpublished observations; but see Tian and Hafed, 2014 for a conference abstract). In any case, we anticipate that neurophysiological tests of this hypothesis will be an area of active research in the near future, and that these tests will allow constraining the space of possible solutions for the question of what mechanisms are behind Posner cueing.

Therefore, both behavioral and neuronal signatures of attention emerge as a function of peri-microsaccadic changes in vision, and theoretical considerations (Figure 8) suggest that such peri-microsaccadic changes can play a significant role in accounting for attentional phenomena. This means that microsaccades may be a key component of the mechanisms of covert visual attention, at least in spatial cueing tasks.

## Conclusion

Over the past several years, both pre- and post-movement neuronal and perceptual consequences of microsaccades have been explored. The main outcome of these studies is that they reveal a real and measurable influence of microsaccades on the state of the visual system. The role of microsaccades in modulating neuronal responses in the visual system is decidedly more *sophisticated* than simple retinal-image refreshing, and it extends to changes in response gain, spatial representations, and potentially neural coding in general. From a practical perspective, this is of broad importance because of the ubiquity of “fixation conditions” in many neuroscience-related experiments using behaving subjects: such experiments often reveal a change in the state of the visual system, but attribute it to other seemingly unrelated phenomena. For example, covert visual attention is—by definition—studied during fixation, and it is thought to alter spatial perception. However, it was found in humans that spatial perception is itself significantly altered before microsaccades (Hafed, 2013). More importantly, such alteration is a significant modulator of behavioral performance changes classically seen in “attentional cueing” experiments (Hafed, 2013), such that oscillations in attentional performance are tightly synchronized to the occurrence of microsaccades. Such tight synchrony with microsaccades even exists for classic *neuronal* signatures of covert visual attention (Chen et al., 2015). These results provide direct demonstration of how the interpretation of classic experiments in systems and cognitive neuroscience may be refined in light of the mechanisms underlying peri-microsaccadic changes in vision that we have reviewed in this article.

One final question that naturally arises out of the work reviewed here is whether the effects that we have summarized so far extend to more ecological conditions outside the laboratory. Specifically, it may be argued that experiments enforcing prolonged gaze fixation are intrinsically unnatural (Poletti and Rucci, 2015). While this may be true, investigating the role of microsaccades in these experiments is in our view still necessary because these experiments themselves often make inferences about vision and cognition in general, and under more ecological conditions. In other words, it is based on such presumably unnatural experiments that general inferences about vision and cognition are often made. Thus, the mechanisms leading to these experiments’ results, including those associated with microsaccades, are still important to unravel. Having said that, it is also fairly simple to envision scenarios in which microsaccades might frequently occur in real life. For example, during high acuity visual tasks, like threading a needle, microsaccades occur frequently in order to precisely relocate the point of gaze (Ko et al., 2010), and they do so because of photoreceptor inhomogeneities within the fovea (Poletti et al., 2013). We think that it is exactly this goal-directed nature of microsaccades in these naturalistic tasks that also drives the great majority of these eye movements during prolonged fixation conditions in the laboratory. Specifically, as far as the oculomotor system is concerned, a primary task in these so-called unnatural experiments is indeed to optimize eye position on the fixation marker, exactly like when threading a needle, and almost irrespective of what the experimenter has designed in the paradigm (e.g., peripheral stimulus discrimination). Coupled with immense foveal magnification in the visual and oculomotor systems, the fixation marker is not necessarily as visually impoverished as one might think, especially when compared with some of the simple stimuli that are often used peripherally (e.g., a landolt C). This sentiment is particularly true in the case of animal studies, because the animals would lose reward if they broke fixation. Thus, a primary component of their task is indeed to optimize eye position on the fixation marker, even though the overwhelming interpretation of experiments is that fixation is a “passive” phenomenon. Given this, we think that the concepts that we have discussed in this review would apply in more natural conditions under which microsaccades would be expected to occur (namely, during high acuity visual tasks). For example, when re-centering the foveola (Poletti et al., 2013), sensitivity would be highest at the microsaccade endpoint but might be suppressed in the opposite direction, and so on for other phenomena that we have summarized in this review.

## Author Contiributions

ZMH, C-YC and XT wrote the manuscript.

## Conflict of Interest Statement

The authors declare that the research was conducted in the absence of any commercial or financial relationships that could be construed as a potential conflict of interest.
